# Effects of the Combined Addition of Zn and Mg on Corrosion Behaviors of Electropainted AlSi-Based Metallic Coatings Used for Hot-Stamping Steel Sheets

**DOI:** 10.3390/ma13153379

**Published:** 2020-07-30

**Authors:** Si On Kim, Won Seog Yang, Sung Jin Kim

**Affiliations:** 1 Department of Advanced Materials Engineering, Sunchon National University, Jungang-ro, Suncheon, Jeonnam 57922, Korea; kzo1102@scnu.ac.kr; 2 R&D Division, Hyundai Steel, 1480 Bukbusaneop-ro, Songak-eup, Dangjin, Chungnam 31719, Korea; wsyang@hyundai-steel.com

**Keywords:** hot stamping steel, AlSi-based coating, electrodeposited paint, Zn, Mg, corrosion

## Abstract

The effects of the combined addition of Zn and Mg on the corrosion resistance of AlSi-based coating for automotive steel sheets were investigated using a variety of analytical and electrochemical techniques. The preferential dissolution of Mg and Zn from MgZn_2_/Mg_2_Si phases occurred on the AlSi-based coating that had been alloyed with a smaller portion of Zn and Mg, which contributed to the rapid surface coverage by corrosion products with a protective nature, reducing the corrosion current density. On the other hand, localized corrosion attacks caused by the selective dissolution of Mg were also observed in the AlSi-based coating with a smaller portion of Zn and Mg. Such alloying can also worsen its corrosion resistance when coated additionally with electrodeposited paint. The mechanistic reasons for these conflicting results are also discussed.

## 1. Introduction

Metallic coatings have been applied widely to steel substrates used in the automotive industry, because of their anti-corrosion performance [1,2,3]. In general, coating materials are more electrochemically active than protected substrates, meaning they offer sacrificial protection. A recent technical issue in a coating system is the formation of suitable corrosion products and new phases that are effective in delaying the coating consumption and reducing the overall corrosion rate [4,5,6]. Regarding Al-based coatings for steel sheets, a passive film (Al_2_O_3_) forms on the coating surface, providing superior barrier protection [7,8,9]. In the case of AlSi-based coatings, the addition of Si decreases the thickness of the intermetallic phase at the inter-diffusion layer close to the steel substrates [10,11], which also provide good anti-corrosion performance [12]. On the other hand, Al_2_O_3_ films are unstable when exposed to environments containing chloride ions (Cl^−^), which can decrease the stability of the film, leading to a decrease in coating efficiency by localized corrosion [13,14]. To overcome these drawbacks, other alloying elements, such as Zn and Mg, which can modify the coating potential, are added to the Al-based coating, so that they can provide a supplementary self-healing effect by the sacrificial dissolution of alloying elements [4,5]. At the same time, several corrosion products acting as a protective barrier can be formed on the outer surface [15,16]. Hence, AlSi-based coatings with Zn and Mg are promising candidates for metallic coatings on hot-stamping steels used for auto-parts [17]. Recently, Nicard et al. [18] examined the anti-corrosion mechanism of AlSi-based coatings with Zn (2~30 wt %) and Mg (1~10 wt %). They reported that the addition of Zn and Mg to AlSi-based coatings increases the corrosion resistance in chloride-containing environments. From a practical and an industrial point of view, however, lower concentrations of Zn and Mg, which have low melting temperatures, are favored to avoid the liquid metal embrittlement (LME) phenomenon [19] and localized corrosion attack caused by the selective dissolution of these alloying elements [6,20].

Furthermore, considering the applicability of the coating to hot-stamped, high strength steel sheets used for auto-body parts, an evaluation of the corrosion resistance should proceed after the electropainting process on the metallic coating. In this regard, this study examined on the corrosion behaviors not only of AlSi-based metallic coating with 10 wt % Zn and 0.5 wt % Mg (refer to AlSiZnMg-MC, here-in-after), but also of electropainted AlSiZnMg-MC. Field-emission scanning electron microscopy (FE-SEM), X-ray diffraction (XRD), X-ray photoelectron spectroscopy (XPS), and potentiostat measurements were used to analyze the corrosion behaviors of metallic coated steels. Moreover, an accelerated corrosion test [12] was conducted on electropainted samples that had been damaged by scratching the coating with an “×” incision, and by colliding the coating surface with fine stone.

## 2. Experimental

### 2.1. Materials and Specimen Preparation

The steel substrate, produced by Hyundai Steel Corp.(Dang Jin, Korea), was classified as 22MnB5 steel with 0.27–0.3 wt % C, 1.3–1.5 wt % Mn, 0.2–0.25 wt % Cr, 0.18–0.2 wt % Si, 0.0025–0.0035 wt % B, 0.02–0.035 wt % Ti. The S and P contents were kept as low as possible to avoid high-temperature cracking during the subsequent stamping process. Two types of metallic coated steel were used: AlSi and AlSi with Zn and Mg (AlSiZnMg) coated steels, which were manufactured using a hot-dip simulator. For the coating process, two types of molten bath were prepared: Al-Si (7 wt %) and Al-Si (7 wt %)-Zn (10 wt %)–Mg (0.5 wt %). The substrates were then dipped in each molten bath, and two types of metallic coating were produced: AlSi-MC and AlSiZnMg-MC, at 50/50 g/m^2^. For the following stamping process, the two metallic coated steels were heated to 930 °C for 300 s and cooled to room temperature by die quenching.

Some of the two types of metallic coated steel sample were also coated with electrodeposited (ED) paint. For this ED coating, the coated steel samples underwent the following processes: degreasing, surface conditioning, and phosphating in a pretreatment simulator. The ED coating was then conducted at 300 V for 180 s while the voltage was increased uniformly for 30 s. The ED coated samples were dried in a circulating oven at 170 °C for 20 min. Detailed information on the ED coating process can be found elsewhere [21]. 

### 2.2. Microstructure Examination

The microstructure and composition of the coated steel samples before and after the corrosion test (potentiostatic (PS) polarization measurement) were examined by FE-SEM and energy dispersive spectroscopy (EDS). Before the corrosion test, the coated steel samples were cleaned ultrasonically in ethanol and dried in air. In particular, for the cross-sectional observations, the samples were mounted with the cut-edge sides facing the surface, and they were polished with a final polishing step of 0.04 μm. After the corrosion test, the sample surface was washed with distilled water and dried in air. The sample was then stored in a vacuum chamber. The various phases in the coating layers were also characterized by XRD.

### 2.3. Depth Profile Analysis

The compositional distributions of the coating layers as a function of depth were examined by glow discharge spectroscopy (GDS) analysis with a Leco (St. Joseph, MI, USA) GDS-850A spectrometer using argon plasma equipped with an RF lamp. The diameter of the analysis area in a sampling area of 20 × 20 mm^2^ was 4 mm.

### 2.4. X-ray Photoelectron Spectroscopy Analysis

XPS was used to analyze the chemical state of the corrosion products formed on the metallic-coated layers after the corrosion test (PS polarization measurement). Prior to analysis, the two types of coated steel sheets that had been corroded by the polarization test were cut into 7 × 8 mm^2^ samples and cleaned using ethanol. XPS (VG Scientific Escalab 250 (Waltham, MA, USA)) was conducted using mono chromatic Al Kα radiation (1486.7 eV) with a 500 μm diameter spot size. A constant analyzer energy mode with 200 and 50 eV for the survey and high-resolution spectra, respectively, were used. Data processing of the spectra was performed using a spectral data processor (SDP) v 3.0 software. The adventitious C 1s peak at 284.8 eV was used as a reference for charge correction [22].

### 2.5. Electrochemical Measurements 

Potentiodynamic and potentiostatic polarization tests were performed after the open circuit potential (OCP) evolution measurements for 1 h in a 3.5% NaCl using a GAMRY (Philadelphia, PA, USA) reference 600. A three-electrode system was used, which consisted of the metallic-coated sample as the working electrode (tested area of 1 cm^2^), a platinum grid as the counter electrode, and a saturated calomel electrode (SCE) as the reference electrode. 

For the potentiodynamic polarization scan, the working electrode was polarized from −0.5 V to 0.25 V vs. OCP at a scan rate of 0.16 mV s^−1^. The corrosion current densities were determined by curve-fitting using the Wagner-Traud equation (Equation (1)), described below, to the potentiodynamic polarization curves.

(1) $$i=i_{corr}\left[\exp\left(\frac{2.303\left(E-E_{corr}\right)}{\beta_{a}}\right)-exp\left(\frac{-2.303\left(E-E_{corr}\right)}{\beta_{c}}\right)\right]$$

where *i*_corr_ is the corrosion current density (A/cm^2^), *E_corr_* and *E* represent the corrosion potential (V) and the measured potential (V), respectively. *β_a_* and *β_c_* are Tafel slopes (V/decade), and *i* is the total corrosion density (A/cm^2^).

Based on the polarization curves, the applied potentials for the anodic and cathodic polarization during the potentiostatic polarization measurements were determined to be 50 mV above the OCP and 100 mV below the OCP, respectively. A preliminary test showed that under an applied potential of 100 mV above the OCP in the potentiostatic polarization, the coating dissolution was too severe to analyze the corrosion behaviors of the coating surface covered with some corrosion products. Therefore, the anodic potential was set to 50 mV lower than the cathodic potential in the polarization measurements. 

Three repetitive tests were conducted for each experiment to ensure reproducibility.

### 2.6. Cyclic Corrosion Test for ED Coated Samples

To evaluate the corrosion resistance of the ED coated AlSi and AlSiZnMg samples, a VDA (Berlin, Germany) 233-102 cyclic corrosion test was conducted according to VDA 233-102 [12], which is a standard for the accelerated corrosion testing of coatings/paintings within the automotive industry. Before the corrosion test, the samples were cut to a size of 150 × 70 mm, and damaged artificially using the following two methods. One set of samples was scratched with an “×” incision in reference to ASTM D-1654 [23], and the other set of samples abraded with a spray of fine stone chips, as reported elsewhere [24]. Salt spraying with a 1% NaCl solution (pH 6.5) at a rate of 2.0 to 4.0 mL/h was then conducted on the samples positioned 65° to the horizontal in an enclosed chamber. A full test cycle lasted for seven days, and the multi-step cycles were composed of varying temperatures ranging from −15 °C to 50 °C and humidity between 50% and 95%. Further information on test conditions can be found in VDA 233-102 [12]. 

## 3. Results and Discussion

### 3.1. Microstructure Characterizations of Metallic Coatings

**Figure 1:**
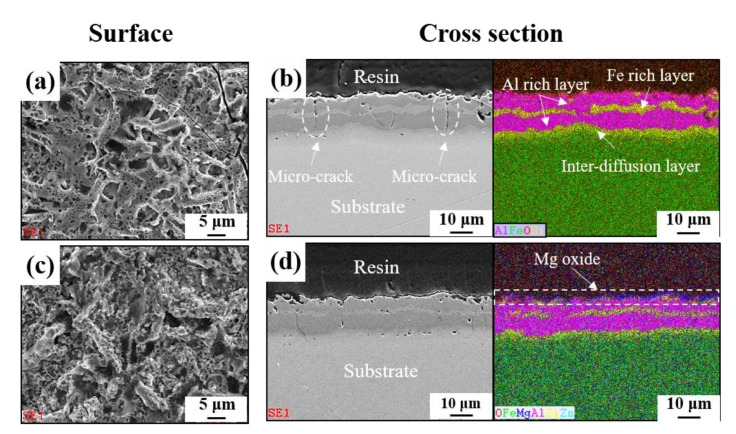
Surface and cross-sectional observations with eds mappings of (**a**,**b**) AlSi-MC and (**c**,**d**) AlSiZnMg-MC.

Figure 1 presents the surface and cross-sectional morphologies and EDS mapping of AlSi-MC (Figure 1a,b) and AlSiZnMg-MC (Figure 1c,d). EDS mapping showed that the two types of coating were composed mainly of Al matrix with two (Al,Fe,Si)-rich intermetallic phases. One of the intermetallic phases formed at the interface between the coating layer and Fe substrate during the hot-dip aluminizing process. The other phases formed in the coating layer during the austenitizing process. The formation of the former phase was attributed to the inter-diffusion of Fe atoms from the steel substrate into the coating and of Al atoms from the coating into the steel substrate [25]. At the same time, Si, known to be concentrated at the outer surface of the steel substrate [26], was also enriched at the inter-diffusion layer. According to previous studies [27,28], the composition of the intermetallic phase is close to τ5 or τ6 shown in the ternary phase diagram of Al-Fe-Si [27,28]. The latter phase was formed by the diffusion of Fe and Si atoms from the steel substrate into the coating during austenitizing for the hot-stamping process, as reported elsewhere [29]. A noticeable feature in the coating morphologies was the presence of micro-cracks and pores. These may be closely associated with the very high hardness (900~1100 HV0.05) of Al-rich Al-Fe intermetallic compounds, such as Al_13_Fe_4_ and Al_5_Fe_2_ [30,31], and the difference in thermal shrinkage among the intermetallic phases during cooling in the hot-stamping process [32]. 

**Figure 2:**
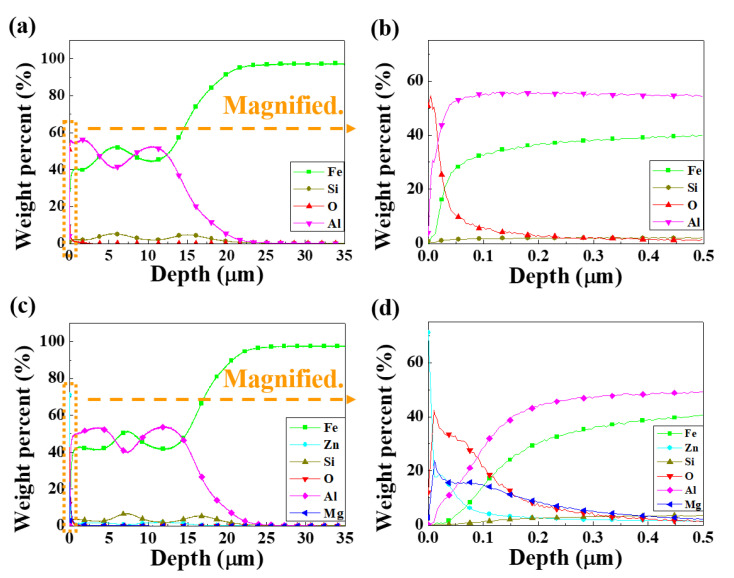
Glow discharge spectroscopy (GDS) depth profile of the coating layers: (**a**,**b**) AlSi-MC, (**c**,**d**) AlSiZnMg-MC.

**Figure 3:**
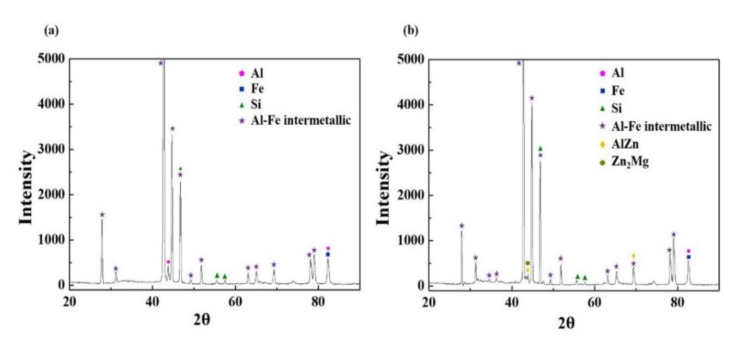
X-ray diffraction patterns of (**a**) AlSi-MC and (**b**) AlSiZnMg-MC.

Although Figure 1 indicated that there were more pores and micro-cracks on the AlSi-MC, the microscopic observations could not be representative of the entire coating layers. On the other hand, the composition features were different apparently in that the outer-surface of AlSiZnMg-MC was covered with a thin (Mg, Zn)—based phase. GDS analysis was conducted for further clarification; Figure 2 presents the depth profile results. In contrast to the surface of AlSi-MC, consisting only of a thin Al-based oxide, the Mg and Zn-based oxide may cover the coating layer of AlSiZnMg-MC. In addition, based on the AlSiZnMg quaternary system [33], Zn-Mg intermetallic phases may exist on the coating surface of AlSiZnMg. For closer analysis, phase characterization was carried out using XRD; the results are shown in Figure 3. As expected, one of the major phases in both coating systems was an Al-Fe intermetallic compound.

Al-Fe, Fe-Si, and Fe-Al-Si intermetallic phases are generally difficult to distinguish by XRD due to overlap of the XRD peaks [12]. The phase existing only in AlSiZnMg-MC was MgZn_2_. The other possible phases in the sample may be Mg_2_Si and MgO. However, the initial fraction of Mg (0.5 wt %) was too low to give prominent peaks of Mg-containing phases in the XRD measurements. Even if some Mg-containing phases were formed, minor fractions that are below the detection limit of the XRD instrument, might be present. Instead of Mg-containing phases, some peaks for the Al-Fe intermetallic phase and Si were detected with low intensity. Considering that the phases, such as MgZn_2_ and Mg_2_Si, have different electrochemical potentials of their own, they can affect the corrosion kinetics and resulting coating life. From an electrochemistry point of view, the phases of MgZn_2_ and Mg_2_Si can act as an anode from the coating matrix because of their lower corrosion potential (−1538 and −1029 mV_SCE_, respectively) [18]. On the other hand, unlike the case of MgZn_2_, which provides sacrificial protection at an early stage of corrosion, Mg_2_Si can form Si clusters after the selective dissolution of Mg, which will be cathodic and cause the localized corrosion attack of an Al-based coating layer. [6,20]. This will be discussed in more detail in the following section.

Another possible phase in AlSiZnMg-MC is MgO, which may be present at the outermost part of the coating surface. Although MgO with low electrical conductivity [34] could contribute in part to the suppression of the cathodic reduction reaction during the early corrosion stage, it is generally non-uniform and, in most cases, increases local corrosion attack. Under this coating composition with a minor fraction of Mg (0.5 wt %), it is extremely difficult to form a stable and uniform MgO film over the coating surface. These discussions were based primarily on the theoretical studies reported elsewhere, and they should be clarified experimentally, which will be discussed in the following section.

### 3.2. Electrochemical Characterizations

**Figure 4:**
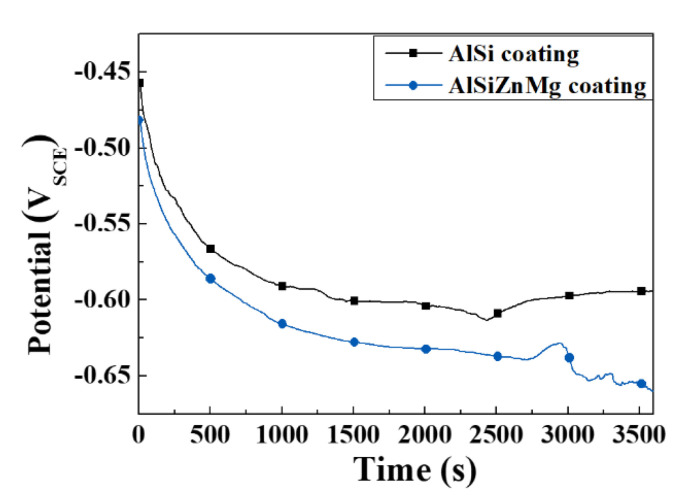
Open circuit potential (OCP) evolution measurement of AlSi-MC and AlSiZnMg-MC for 1 h in a 3.5% NaCl solution [20].

The evolution of the OCP (Figure 4) was measured for 1 h before the electrochemical polarization tests. The coating potentials of AlSi-MC and AlSiZnMg-MC after 1 h of immersion were approximately −0.59 V_SCE_, and −0.62 V_SCE_, respectively. In contrast to the Al coating covered by a thin Al oxide film (Al_2_O_3_), Zn and Mg, which were enriched at the coating surface, rarely form an oxide/hydroxide film with a high passivity coefficient in a neutral solution, which could result in a lower corrosion potential. As mentioned previously, the presence of active phases, such as Mg_2_Si and MgZn_2_, can contribute to the decrease in corrosion potential. On the other hand, the preferential dissolution of Mg from the active phases can provide sacrificial protection. Previous studies [16,35] have shown that Mg and Zn cations, which are supplied by the selective dissolution of MgZn_2_ (reaction (2)), can also lead to rapid coverage of the coating surface by the precipitation of corrosion products with a protective nature, such as simonkolleite (Zn_5_(OH)_8_Cl_2_·H_2_O), which can prevent further corrosion.

MgZn_2_(s)→Mg^2+^(aq) + 2Zn^2+^(aq) + 6e^−^ (2)

According to a previous study, the selective dissolution of Mg from the Mg_2_Si phase can provide cathodic protection. They proposed that the formation of less conductive SiO_2_ can decrease the galvanic current between Al/SiO_2_ according to reaction (3) [18,36]:

Mg_2_Si(s) + 2H_2_O(l)→2Mg^2+^(aq) + SiO_2_(s) + 4H^+^(aq) + 8e^−^ (3)

On the other hand, the formation of a stable SiO_2_ film uniformly formed on the coating surface cannot be guaranteed. Under this condition, an unstable film may create active local galvanic coupling, increasing the dissolution of the coating layer.

**Figure 5:**
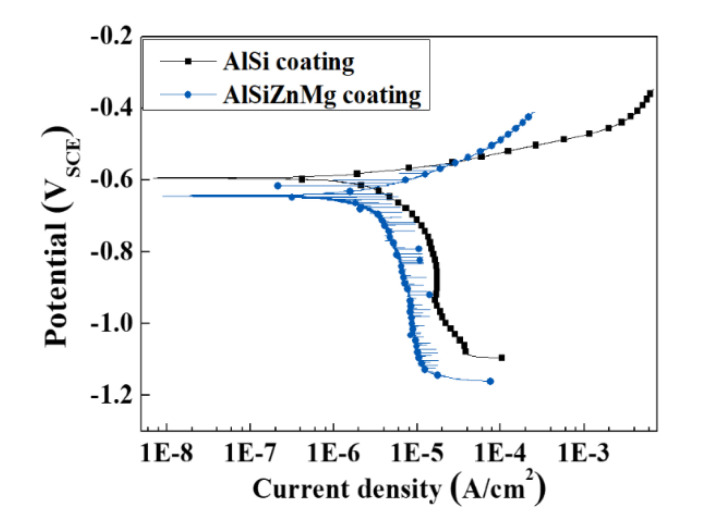
Potentiodynamic polarization curves of AlSi-MC and AlSiZnMg-MC, measured after 1 h immersion in a 3.5% NaCl solution [20].

**Figure 6:**
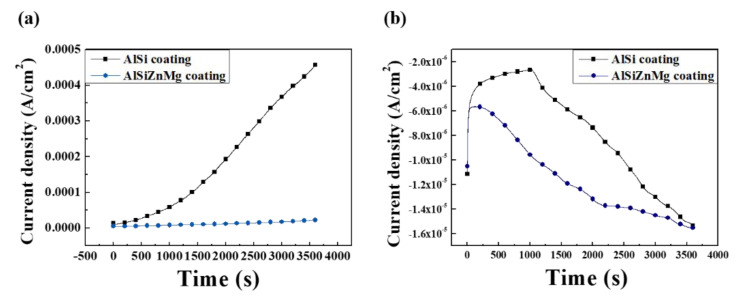
Potentiostatic polarization curves of AlSi-MC and AlSiZnMg-MC, measured after 1 h immersion in a 3.5% NaCl solution under (**a**) anodic (50 mV above OCP), and (**b**) cathodic (100 mV below OCP) potentials [20].

**Figure 7:**
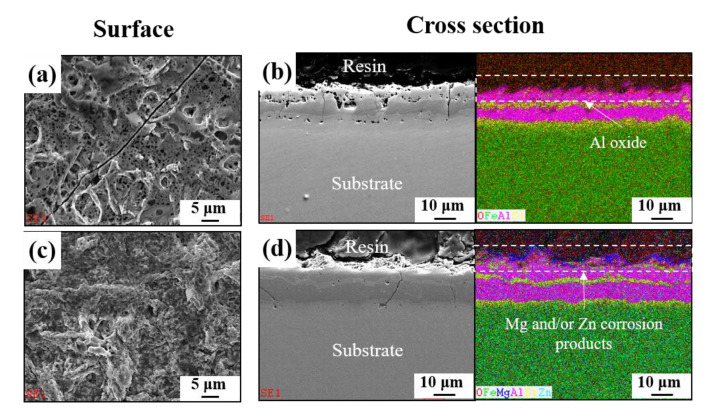
Surface and cross-sectional observations with EDS mappings of (**a**,**b**) AlSi-MC and (**c**,**d**) AlSiZnMg-MC after the potentiostatic (PS) test.

These beneficial and harmful effects on the corrosion resistance can be examined by polarization measurements, which are shown in Figure 5; Figure 6. Potentiodynamic and potentiostatic polarizations showed that the anodic and cathodic reaction rates of AlSiZnMg-MC were much smaller than those of AlSi-MC. The corrosion current densities (*i_corr_*) determined by curve-fitting with the Wagner–Traud equation to the polarization curves of AlSiZnMg-MC and AlSi-MC were 3.5 and 8.5 µA cm^−2^, respectively. The lower anodic and cathodic current densities of AlSiZnMg-MC suggest that the liberated Mg^2+^ and Zn^2+^ ions, supplied by the selective dissolution of Mg and Zn, provided an environment for the formation of corrosion products contributing to the effective reduction of the anodic and cathodic current densities. On the other hand, the polarization curve of AlSiZnMg-MC has many fluctuations, which may be closely associated with localized corrosion caused by the non-uniform distribution of MgO or the selective dissolution of Mg from Mg_2_Si phases in the coating. This can be supported in part by several local corrosion attacks after the corrosion test, as shown in Figure 7. From morphological observations before (Figure 1) and after (Figure 7) the corrosion test (PS polarization), there was little difference except that the outermost surfaces were less uniform after the corrosion test. Compared to the case of AlSi-MC, the surface morphology of AlSiZnMg-MC after the corrosion test appeared to be denser (Figure 7c). On the other hand, AlSiZnMg-MC exhibited a more uneven cross-sectional view, which may be associated with the formation of corrosion products containing Mg, Zn, and Si, and localized corrosion attacks mentioned previously. Owing to the low resolution of EDS or GDS, XPS was performed to characterize the corrosion products formed on the two types of coating after the corrosion test.

### 3.3. Characterization of the Corrosion Products by XPS

**Figure 8:**
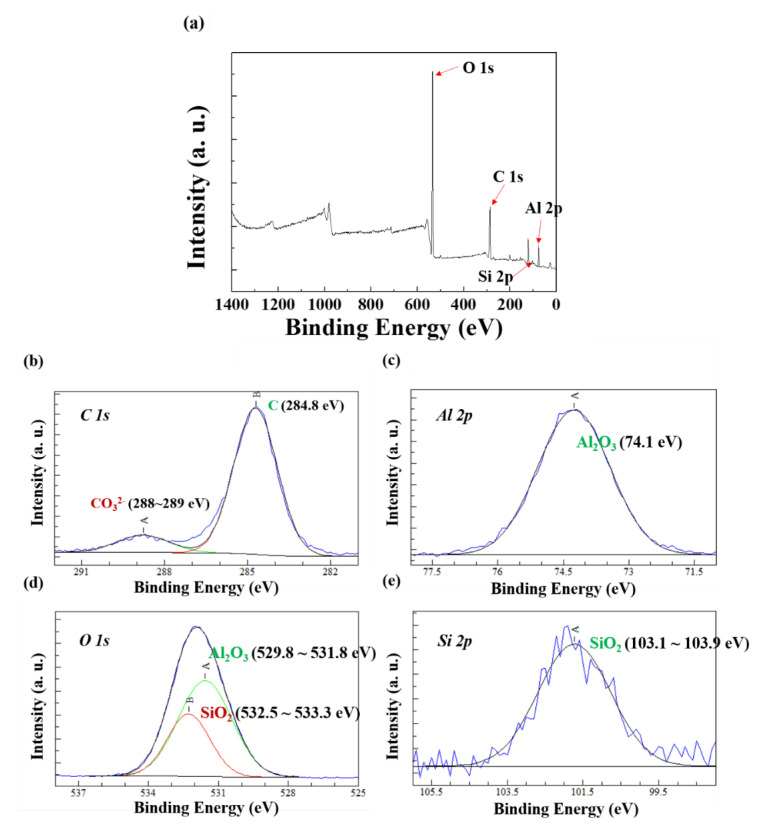
(**a**) XPS survey spectrum and (**b**–**e**) high-resolution spectra of the surface on AlSi-MC after the PS test.

**Figure 9:**
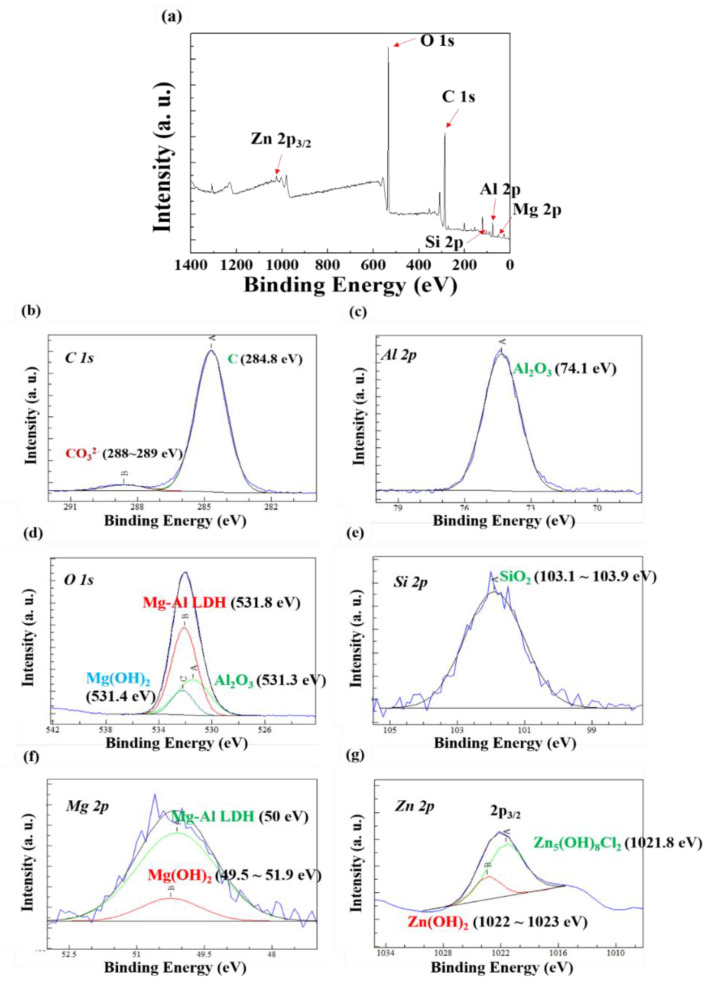
(**a**) XPS survey spectrum and (**b**–**g**) high-resolution spectra of the surface on AlSiZnMg-MC after PS test.

Figure 8 presents the XPS survey spectrum of the surface of AlSi-MC. The spectrum of the coating surface revealed C 1s, Al 2p, O 1s, and Si 2p primarily. The high-resolution spectra, shown in Figure 8c–e, indicated that the corrosion products formed on the surface were composed mainly of Al_2_O_3_ with a smaller portion of SiO_2_. On the other hand, the survey spectrum of the surface on AlSiZnMg-MC (Figure 9) showed that, in addition to C 1s, Al 2p, O 1s, and Si 2p, Mg 2p and Zn 2p were observed on the coating surface, and the oxygen intensity was higher. This suggests that Zn and Mg-based oxide/hydroxide are major components in the coating surface. From the high-resolution spectra of the surface on AlSiZnMg-MC, as shown in Figure 9c–g, the coating surface was composed of a wider variety of corrosion products including Al_2_O_3_, Mg(OH)_2_, SiO_2_, Zn(OH)_2_, Zn_5_(OH)_8_Cl_2_·H_2_O, and Mg-Al layered double hydroxide (LDH) with the general formula, M_x_Al_y_(A)_m_(OH)_n_._z_H_2_O (where A and M represent an anion and a di-valent cation, respectively) [16,18,37]. As mentioned previously, the addition of Zn and Mg to the coating can help increase the corrosion resistance in a neutral aqueous solution, in such a way that several corrosion products with an inhibiting nature are formed preferentially on the surface at the early stages of corrosion. Among the products, Mg-Al LDH and Zn_5_(OH)_8_Cl_2_·H_2_O can act as effective barriers for oxygen diffusion [38,39] and provide superior corrosion resistance in neutral aqueous solutions [38,39]. On the other hand, the formation or dissolution of other products can also help increase the corrosion resistance by stabilizing Mg-Al LDH and Zn_5_(OH)_8_Cl_2_·H_2_O. First, the formation of Mg-Al LDH, as shown in Figure 9d,f involves reactions (2), (4), (5), and (6).

Oxygen reduction:

O_2_(g) + 2H_2_O(l) + 4e^−^→4OH^−^(aq) (4)

Dissolution of alumina:

Al_2_O_3_(s) + 3H_2_O(l) + 2OH^−^(aq)→2Al(OH)^4−^(aq) (5)

Dissolution of metallic Al:

Al(s) + 4OH^−^(aq)→Al(OH)^4−^(aq) + 3e^−^ (6)

Reactions (5) and (6) can occur locally at the cathode area because of the instability of Al/Al_2_O_3_ at alkaline pH [16]. As a result, the formation reaction of LDH can proceed, as described below:

2Al(OH)^4−^(aq) + 6Mg^2+^(aq) + 8OH^−^(aq) + CO_3_^2−^(aq)→Mg_6_Al_2_(OH)_16_CO_3_(s) (7)

The stability of LDH is dependent on the pH [40,41], and LDH can dissolve under weakly alkaline condition. On the other hand, the dissolution of LDH can be suppressed by the formation of a thin Mg(OH)_2_ layer (reaction (8)), called the skin effect [15,37]:

Mg^+^(aq) + 2OH^−^(aq)→Mg(OH)_2_(s) (8)

Second, the formation of Zn_5_(OH)_8_Cl_2_·H_2_O, as shown in Figure 9g, can be described below:

4ZnO(s) + Zn^2+^(aq) + 5H_2_O(l) + 2Cl^−^(aq)→Zn_5_(OH)_8_Cl_2_·H_2_O(s) (9)

The presence of Mg^2+^ supplied by reaction (2) can also delay the following reaction, which leads to slower dissolution kinetics of Zn_5_(OH)_8_Cl_2_·H_2_O. The formations of Mg-Al LDH and Zn_5_(OH)_8_Cl_2_·H_2_O with high stability are the major mechanistic reasons for the higher corrosion resistance of AlSiZnMg in a neutral environment containing Cl^−^. Nevertheless, the increased possibility of local corrosion of the AlSiZnMg sample cannot be excluded. In addition, its superiority and utility as a coated steel should also be evaluated after the electropainting process, which will be discussed in the following section.

### 3.4. Surface Characteristics after Electropainting

**Figure 10:**
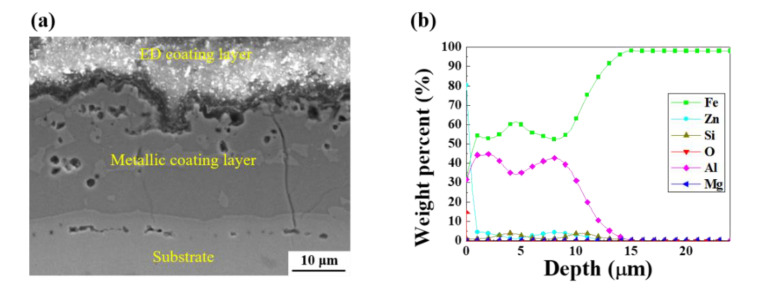
(**a**) Cross-sectional observation and (**b**) GDS depth profile of ED-treated AlSiZnMg-MC.

Figure 10 presents the GDS depth profiles of AlSi-MC and AlSiZnMg-MC after the ED coating. In contrast to AlSi-MC, Zn(Mg)—based oxides were present on the outer surface of the ED-coated AlSiZnMg-MC. This suggests that the oxides remained on the outer surface, even after surface cleaning before the ED coating. As mentioned in the previous section, a stable thick MgO film cannot be formed in a minor fraction of Mg (0.5 wt %) in this coating system. An unstable oxide film on the surface may have weakened the adhesion between the inner metallic coating/outer ED coating and reduced the corrosion resistance.

### 3.5. Corrosion Resistance Evaluations after Electropainting

**Figure 11:**
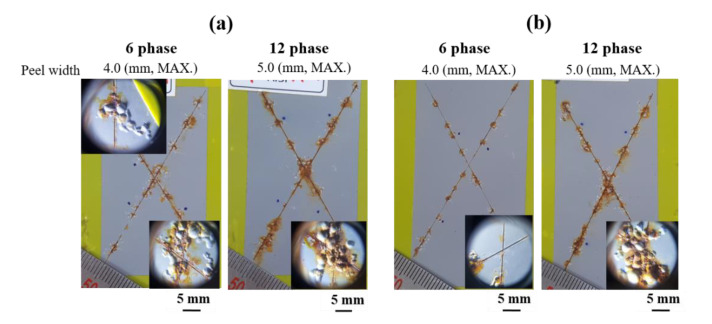
Surface appearance of electrodeposited (ED)-treated (**a**) AlSi-MC and (**b**) AlSiZnMg-MC, which had been scratched with an “×” incision, after the accelerated corrosion test.

**Figure 12:**
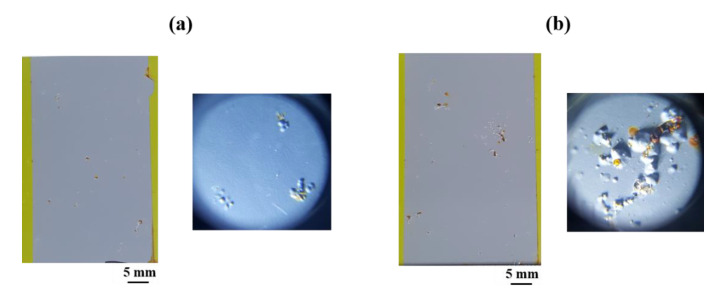
Surface appearance of ED-treated (**a**) AlSi-MC and (**b**) AlSiZnMg-MC, which had been collided with fine stone chips, after the accelerated corrosion test.

Figure 11 shows the surface appearance of the ED-treated AlSi-MC (Figure 11a) and AlSiZnMg-MC (Figure 11b), after the accelerated corrosion test in reference to VDA 233-102 [12]. Considering the many test results presented above, it was expected that ED-treated AlSiZnMg-MC showed higher corrosion resistance when evaluated by an accelerated corrosion test. After the ED coating, however, its higher corrosion resistance was not observed clearly in that the damaged areas and extent of red rust formation on the scribed regions with an X between the two coated steels sheets became similar as the number of cycles increased. In addition, the surface morphologies of the two ED-coated steel samples that had been subjected to stone chipping and a subsequent corrosion test revealed even more severe surface degradation of ED-coated AlSiZnMg-MC. As shown in Figure 12, the size and number of surface blisters were larger on the ED-coated AlSiZnMg-MC. These blisters resulted from volumetric expansion caused by the formation of corrosion products on the steel substrate covered with a metallic coating layer. This suggests that the corrosive species are more able to permeate through the ED coating on AlSiZnMg-MC, and the electrochemical corrosion reactions occurred rapidly. The higher degradation by the corrosion reactions of ED coated AlSiZnMg-MC may be associated closely with the lower adhesion between the inner metallic coating/outer ED coating. As mentioned previously, the (Zn, Mg)-based oxide formed on AlSiZnMg can weaken the adhesion between the inner/outer coating layers, leading to delamination of the outer coating layer when attacked mechanically and chemically. This is supported by the lower coefficient of friction for the ED coating on AlSiZnMg-MC, which was measured by surface and interfacial cutting analysis, and was reported elsewhere [21]. Hence, the addition of Zn and Mg to the AlSi-based metallic coating does not always have beneficial effects on the corrosion resistance when the metallic-coated steels are coated additionally with ED paint. These results provide useful insights into the development of coated steel sheets used in automotive industries. Nevertheless, further technical research on optimizing the compositions of Zn and/or Mg alloying that provides sacrificial corrosion protection to the AlSi coated steel while reducing the possibility of local corrosion and securing superior corrosion resistance even after additional ED coating will be needed.

## 4. Conclusions

The influence of the combined addition of Mg and Zn on the microstructure and corrosion resistance of AlSi-based coatings for steel sheets was investigated using a range of analytical and experimental techniques. In particular, the corrosion resistance was also evaluated after ED coating on the metallic coated steel sheets. The major findings can be summarized as follows:

1. The two types of AlSi-based coating (AlSi-MC and AlSiZnMg-MC) were composed mainly of an Al matrix with two (Al,Fe,Si)-rich intermetallic phases. The compositional features were different in that the intermetallic phases of MgZn_2_ and Mg_2_Si existed only in AlSiZnMg-MC covered with a thin outermost layer of (Zn, Mg)-based oxide.
2. The active phases formed in AlSiZnMg-MC, such as Mg_2_Si and MgZn_2_, can decrease the corrosion potential. The preferential dissolution of Mg and Zn from the active phases can lead to rapid coverage of the coating surface by the precipitation of corrosion products with a protective nature, resulting in a much smaller current density for both anodic and cathodic reactions. On the other hand, the polarization curve of AlSiZnMg-MC showed many fluctuations, which may be closely associated with localized corrosion caused by the non-uniform distribution of MgO or the selective dissolution of Mg from Mg_2_Si phases in the coating.
3. Compared to the case of the AlSi-MC, XPS showed that the surface of AlSiZnMg-MC was composed of a wider variety of corrosion products, including Al_2_O_3_, Mg(OH)_2_, SiO_2_, Zn(OH)_2_, Zn_5_(OH)_8_Cl_2_·H_2_O, and Mg-Al LDH. Among the products, the presence of Zn_5_(OH)_8_Cl_2_·H_2_O and Mg-Al LDH, stabilized by Mg(OH)_2_, is the major mechanistic reason for the higher corrosion resistance of AlSiZnMg-MC.
4. In contrast to the corrosion resistance of metallic coatings, ED-coated AlSiZnMg-MC exhibited more severe surface degradation after the accelerated corrosion tests. The non-uniform formation of Mg-based oxide over the AlSiZnMg-MC can weaken the adhesion between the inner metallic coating/outer ED coating. Hence, the corrosive species are more able to permeate through the ED coating on AlSiZnMg-MC, and the electrochemical corrosion reactions occurred rapidly. Therefore, further optimization of the alloy contents in AlSi-based coatings needs to be investigated.
